# Evolution of Blood-Brain Barrier Permeability in Subacute Ischemic Stroke and Associations With Serum Biomarkers and Functional Outcome

**DOI:** 10.3389/fneur.2021.730923

**Published:** 2021-10-20

**Authors:** Sarah Müller, Anna Kufner, Andrea Dell'Orco, Torsten Rackoll, Ralf Mekle, Sophie K. Piper, Jochen B. Fiebach, Kersten Villringer, Agnes Flöel, Matthias Endres, Martin Ebinger, Alexander H. Nave

**Affiliations:** ^1^Center for Stroke Research Berlin, Charité - Universitätsmedizin Berlin, Corporate Member of Freie Universität Berlin, Humboldt-Universität zu Berlin, Berlin Institute of Health, Berlin, Germany; ^2^Klinik und Hochschulambulanz für Neurologie - Department of Neurology, Charité - Universitätsmedizin Berlin, Berlin, Germany; ^3^Berlin Institute of Health (BIH), Berlin, Germany; ^4^BIH QUEST - Center for Transforming Biomedical Research, Berlin Institute of Health (BIH), Berlin, Germany; ^5^ExcellenceCluster NeuroCure, Charite-Universitätsmedizin Berlin, Corporate Member of Freie Universität Berlin, Humboldt-Universität zu Berlin and Berlin Institute of Health, Berlin, Germany; ^6^Institute of Biometry and Clinical Epidemiology, Charité - Universitätsmedizin Berlin, Berlin, Germany; ^7^Department of Neurology, University Medicine Greifswald, Greifswald, Germany; ^8^German Center for Neurodegenerative Diseases (DZNE), Rostock/Greifswald, Germany; ^9^German Centre for Cardiovascular Research (DZHK), Berlin, Germany; ^10^German Center for Neurodegenerative Diseases (DZNE), Berlin, Germany; ^11^Department of Neurology, Medical Park Berlin Humboldtmühle, Berlin, Germany

**Keywords:** ischemic stroke, subacute, biomarkers, blood-brain barrier, functional outcome

## Abstract

**Background and Purpose:** In the setting of acute ischemic stroke, increased blood-brain barrier permeability (BBBP) as a sign of injury is believed to be associated with increased risk of poor outcome. Pre-clinical studies show that selected serum biomarkers including C-reactive protein (CRP), interleukin-6 (IL-6), tumor necrosis factor-alpha (TNFα), matrix metallopeptidases (MMP), and vascular endothelial growth factors (VEGFs) may play a role in BBBP post-stroke. In the subacute phase of stroke, increased BBBP may also be caused by regenerative mechanisms such as vascular remodeling and therefore may improve functional recovery. Our aim was to investigate the evolution of BBBP in ischemic stroke using contrast-enhanced (CE) magnetic resonance imaging (MRI) and to analyze potential associations with blood-derived biomarkers as well as functional recovery in subacute ischemic stroke patients.

**Methods:** This is an exploratory analysis of subacute ischemic stroke patients enrolled in the *BAPTISe* study nested within the randomized controlled *PHYS-STROKE* trial (interventions: 4 weeks of aerobic fitness training vs. relaxation). Patients with at least one CE-MRI before (v1) or after (v2) the intervention were eligible for this analysis. The prevalence of increased BBBP was visually assessed on T1-weighted MR-images based on extent of contrast-agent enhancement within the ischemic lesion. The intensity of increased BBBP was assessed semi-quantitatively by normalizing the mean voxel intensity within the region of interest (ROI) to the contralateral hemisphere (“normalized CE-ROI”). Selected serum biomarkers (high-sensitive CRP, IL-6, TNF-α, MMP-9, and VEGF) at v1 (before intervention) were analyzed as continuous and dichotomized variables defined by laboratory cut-off levels. Functional outcome was assessed at 6 months after stroke using the modified Rankin Scale (mRS).

**Results:** Ninety-three patients with a median baseline NIHSS of 9 [IQR 6–12] were included into the analysis. The median time to v1 MRI was 30 days [IQR 18–37], and the median lesion volume on v1 MRI was 4 ml [IQR 1.2–23.4]. Seventy patients (80%) had increased BBBP visible on v1 MRI. After the trial intervention, increased BBBP was still detectable in 52 patients (74%) on v2 MRI. The median time to v2 MRI was 56 days [IQR 46–67]. The presence of increased BBBP on v1 MRI was associated with larger lesion volumes and more severe strokes. Aerobic fitness training did not influence the increase of BBBP evaluated at v2. In linear mixed models, the time from stroke onset to MRI was inversely associated with normalized CE-ROI (coefficient −0.002, Standard Error 0.007, *p* < 0.01). Selected serum biomarkers were not associated with the presence or evolution of increased BBBP. Multivariable regression analysis did not identify the occurrence or evolution of increased BBBP as an independent predictor of favorable functional outcome post-stroke.

**Conclusion:** In patients with moderate-to-severe subacute stroke, three out of four patients demonstrated increased BBB permeability, which decreased over time. The presence of increased BBBP was associated with larger lesion volumes and more severe strokes. We could not detect an association between selected serum biomarkers of inflammation and an increased BBBP in this cohort. No clear association with favorable functional outcome was observed.

**Trial registration:** NCT01954797.

## Introduction

Increased blood-brain barrier permeability (BBBP) is frequently observed after ischemic stroke and can be a sign of acute injury. In the subacute setting, increased BBBP may be a result of recovery mechanisms including neuroprotective inflammation and angiogenesis. Bernardo-Castro et al. and Yang et al. previously summarized the main pathophysiological processes likely underlying the evolution of BBBP in subacute stages post-stroke (1, 2). Directly following an acute vessel occlusion, oxidative stress and subsequent inflammation lead to early impairment of the blood-brain barrier (BBB). Subsequent reperfusion and endothelial damage lead to further BBB impairment (3) and can last up to weeks following the index event (4). The underlying pathophysiology of increased BBBP is highly complex (5, 6) and may play a crucial role in tissue recovery and outcome post-stroke. However, the exact time course and role of selected biomarkers in increased BBBP following ischemia remain poorly understood.

In the acute phase, both the ischemic event and subsequent reperfusion can result in oxidative stress and neuro-inflammation of the brain (1). The inflammatory processes result in increased cytokine levels such as interleukin-6 (IL-6), tumor necrosis factor-alpha (TNFα), and the acute phase reactant C-reactive protein (CRP) (7–9). Preclinical studies suggest that selected pro-inflammatory proteins and proteolytic enzymes may play a causal role in increased BBBP by negatively modifying tissue recovery following cerebral ischemia (10–13). Clinical imaging studies in stroke patients found high matrix metallopeptidases-9 (MMP-9) blood levels to be associated with secondary brain damage in the hyper-acute and acute stage after an ischemic stroke (14, 15). Animal studies with histological assessment of rat brains suggest that high MMP-9 expression in histological samples have a negative effect on BBB function and ultimately post-stroke recovery (16). In the acute setting of an ischemic stroke, the upregulation of vascular endothelial growth factor (VEGF) was observed to influence the BBB integrity by increasing para-cellular permeability (17, 18). Moreover, using magnetic resonance imaging (MRI) of rodent brains, Zhang et al. could show that intravenous application of VEGF may enhance angiogenesis in the ischemic penumbra during the subacute phase of an IS and hence potentially modify outcome (19).

Stroke rehabilitation studies hypothesize that through the reduction of oxidative stress and anti-inflammatory processes, aerobic fitness training may improve BBB integrity (20). In line with this hypothesis, previous studies suggest that physical training post-stroke may promote synaptic plasticity and enhance neurogenesis and angiogenesis, leading to better functional recovery (21–23).

Both the underlying mechanisms of increased vascular remodeling and angiogenesis were observed to be associated with better functional recovery following ischemia in rodent studies (24). Previously published clinical studies using imaging techniques (including MRI and single photon emission computed tomography) found that increased BBBP in hyperacute and acute phases of ischemic stroke was associated with poor functional outcome (14, 25, 26). There are still no clinical studies published investigating potential associations of increased BBBP and functional recovery in subacute ischemic stroke patients. However, a comprehensive analysis of how selected blood serum biomarkers and aerobic physical training may affect BBBP post-stroke in the clinical setting is still lacking.

In this study, we aimed to assess the evolution of BBBP in subacute ischemic stroke assessed via contrast-enhanced MRI using the prospective *BAPTISe* study (“Biomarkers and Perfusion—Training-Induced changes after Stroke”) that accompanied the randomized controlled stroke rehabilitation trial *PHYS-STROKE* (“Physical Fitness Training in Patients with Subacute Stroke”). In this exploratory analysis, we evaluated underlying associations of increased BBBP with selected blood biomarkers, exposure to early aerobic fitness training as well as long-term functional recovery after stroke.

## Materials and Methods

### Study Design

All patients were enrolled in the prospective, observational *BAPTISe* study (27) nested within the multicenter, randomized-controlled *PHYS-STROKE* trial (28). In *PHYS-STROKE*, 200 subacute stroke patients were randomized to a 4-week intervention of aerobic bodyweight supported, treadmill-based physical fitness training vs. relaxation sessions. All participants provided written informed consent to participate in this study. The study was approved by the institutional review board of Charité–Universitätsmedizin Berlin (EA1/138/13). The trial did not show a significant difference in the co-primary efficacy endpoints: maximal walking speed and Barthel Index at 3 months after stroke. A detailed description of the trial intervention, outcome assessments, and the main analyses of the *PHYS-STROKE* trial were reported previously (29).

**Figure 1:**
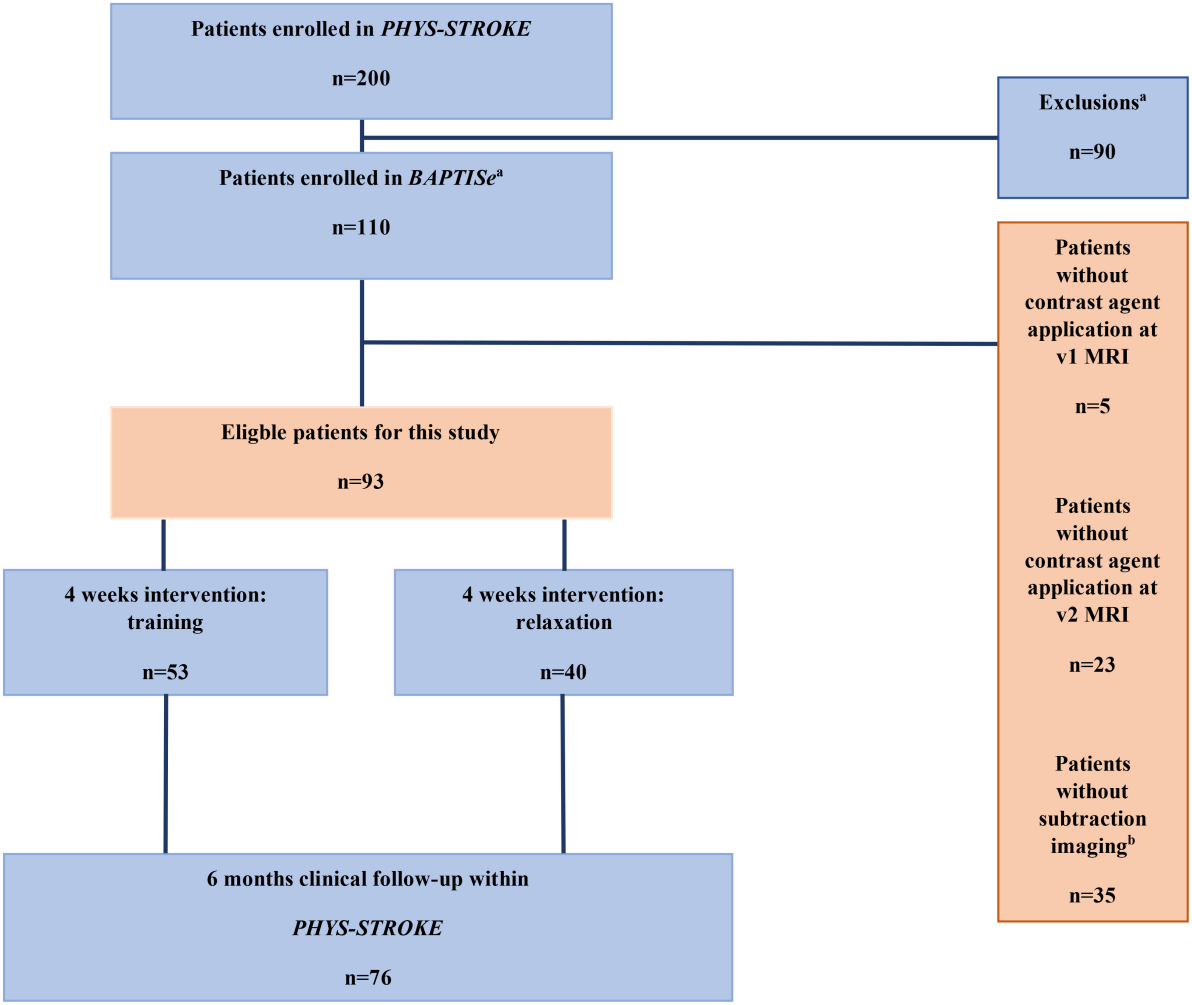
Study flow chart. ^a^Exclusion criteria for *BAPTISe* study are listed in Supplementary Table 1. ^b^Exclusions due to complex post-processing steps.

All patients enrolled in *BAPTISe* had a subacute ischemic stroke (5–45 days after stroke onset) and received cerebral MRI before and after the trial intervention. The time of stroke onset for each patient was documented based on written reports from the primary treating stroke unit and confirmed by patients and/or relatives. The main inclusion criteria of the *BAPTISe* study are listed in Supplementary Table 1. Patients who received at least one MRI with contrast agent application were eligible for this analysis. Inclusion and exclusion criteria are depicted in the study flow chart (Figure 1).

### Clinical and Blood Biomarker Assessment

Patient demographics including medical history, treatment with systemic thrombolysis, Trial of ORG 10172 in Acute Stroke Treatment (TOAST) classification, and National Institute of Health Stroke Scale (NIHSS) at the primary treating stroke unit were documented. The NIHSS was additionally assessed at each MRI visit of the *BAPTISe* study before (v1) and after (v2) the intervention. Clinical follow-up took place at 3 and 6 months after stroke. To assess long-term functional outcome, we used the modified Rankin Scale (mRS) at 6 months post-stroke. A favorable outcome was defined as an mRS score of 0–2. In additional exploratory analyses, we defined an independent functional outcome at an mRS of 3, which reflects the median split of our cohort.

The following blood biomarkers were assessed prior to the start of the trial intervention at v1: high sensitive C-reactive protein (hs-CRP), IL-6, TNF-α, MMP-9, and VEGF. The biomarkers hs-CRP, TNF-α, and IL-6 were directly measured by solid-phase, chemiluminescent immunometric assays (IMMULITE® 1000, Siemens Healthcare Diagnostics) within 6 h after blood draw. Both MMP-9 and VEGF were analyzed from a subsample of patients by enzyme-linked immunosorbent assay (ELISA) in serum samples after being frozen at −80°C. The following laboratory cut-off levels were used to define elevated serum levels: hs-CRP ≥3.0 mg/L, IL-6 ≥3.6 pg/ml, TNF-α ≥8.1 pg/ml, VEGF ≥991 pg/ml, and MMP-9 ≥1,279 ng/ml.

All MRI examinations before (v1) and after (v2) the trial intervention were performed on a 3-Tesla MRI scanner (TIM Trio; Siemens AG, Erlangen, Germany). The MRI protocol of *BAPTISe* was previously published (27). Increased permeability of the BBB was assessed on T1-weighted images following intravenous administration of contrast agent (0.13 ml/kg body weight of Gadolinium).

### Qualitative and Semi-quantitative Assessment of Increased BBBP

**Figure 2:**
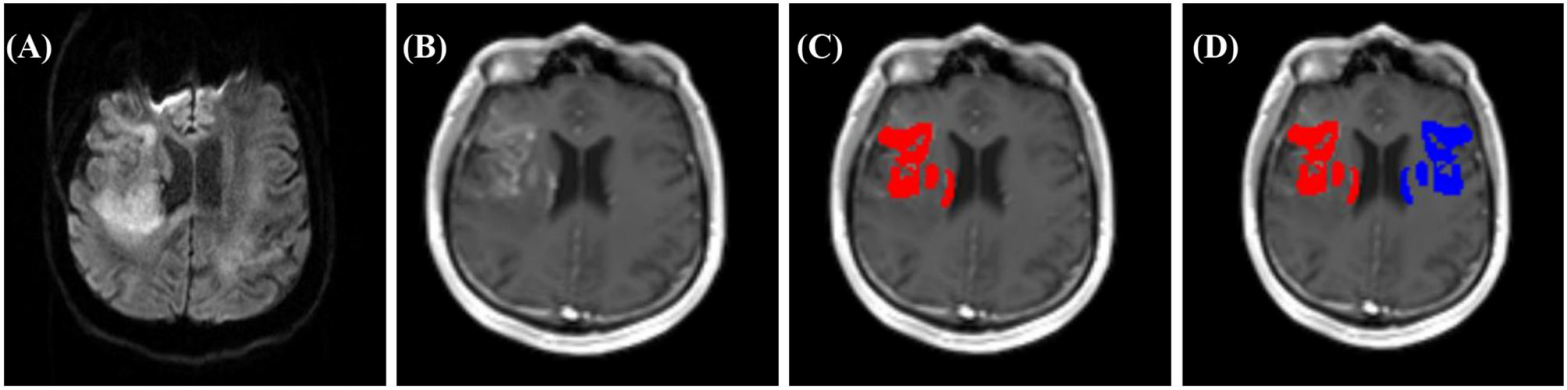
Example of BBBP assessment in a patient with subacute right middle cerebral artery infarction. **(A)** DWI b1000 for the detection of the infarcted area, **(B–D)** T1 post-CA sequence: **(B)** with contrast-agent enhancement (CE), **(C)** delineation of CE within ischemic lesion (CE-ROI red), with **(D)** corresponding mirrored ROI (blue).

The presence of increased BBBP was qualitatively assessed and defined as a visual contrast-agent enhancement (CE) within the ischemic lesion on T1-weighted sequences (30–32). First the infarcted area was identified on diffusion weighted imaging (DWI; b1000), as presented in Figure 2A. Corresponding, T1-weighted sequences after contrast agent application were analyzed and assessed for CE within the area of diffusion restriction as presented in Figure 2B. The region with CE was defined as the region of interest (CE-ROI) and manually delineated by one experienced rater (S.M.). The CE-ROI was subsequently mirrored to the contralateral healthy hemisphere (mirrored ROI, see Figures 2C,D).

The intensity of increased BBBP was assessed semi-quantitatively by normalizing the mean voxel intensity of the CE-ROI to the mean voxel intensity of the mirrored ROI and defined as the normalized CE-ROI as depicted in Figure 2.

### Subtraction Imaging: Evolution of BBBP

For visualization and qualitative evaluation of the evolution of BBBP within one subject, we used T1-weighted subtraction images. T1-weighted images before and after contrast agent application (pre- and post-contrast agent) injection were aligned and co-registered at v1 and v2 with the previously described ROI using FSL (FMRIB Software Library v6.0; https://fsl.fmrib.ox.ac.uk/fsl/fslwiki/Fslutils) and ANTs (Advanced Normalization Tools; https://stnava.github.io/ANTs/) software. First, the pre—post-contrast agent image at v1 was subtracted from the pre-post-contrast agent image at v2. Subsequently, the evolution of BBBP was qualitatively assessed on the resulting final subtraction images: a visible hyper-intense signal was defined as an increase of the BBBP and a hypo-intense signal as a decrease of BBBP over time. The absence of intensity changes was categorized as an unchanged BBBP.

### Statistical Analysis

Continuous variables with skewed distribution are reported as a median with interquartile range (IQR); those with normal distribution are reported as means with standard deviations (SDs). We used the Student's *t*-test and Wilcoxon–Mann Whitney–*U*-test to evaluate the difference between groups of continuous variables. Categorical variables are presented as frequency and percentage. We compared categorical variables by using the chi-squared test.

In order to assess parameters associated with increased BBBP intensity on v1 and v2 MRIs, we performed linear mixed-model analyses with normalized lesion-CE as the dependent variable. The subject was included as a random effect. Fixed effects included time points of MRI (v1 vs. v2), time to MRI in days, and intervention group. In a second linear mixed-model analysis, the model was adjusted for parameters that reached significance (*p* < 0.05) in univariate analysis.

Furthermore, we performed logistic regression analysis to evaluate the association between increased BBBP and patient characteristics and blood biomarkers. The prevalence and evolution of increased BBBP were analyzed as dependent variables. We assessed the association of both the prevalence and evolution of increased BBBP and the dependent variable of favorable outcome at 6 months after stroke using multivariable logistic regression analyses: models were adjusted for age, sex, and variables that reached a significance of *p* ≤ 0.1 in univariate analyses.

Due to the exploratory approach of this study, we did not correct for multiple testing. The significance level was defined as *p* ≤ 0.05. All *p*-values constitute exploratory research and do not allow for confirmatory generalization of results. All statistical analyses were performed using SPSS Version 25 for Windows (SPSS Inc.) and Stata/IC 14.1 for Windows (StataCorp LP).

## Results

### Prevalence, Intensity, and Evolution of Increased BBBP

Of 110 patients included in the *BAPTISe* study, 85% (*n* = 93) met the criteria of at least one MRI-scan with contrast agent application. Thirty-eight percent of the participants were women, the mean age was 68.5 years (*SD* 11.3), and the median acute NIHSS assessed in the primary treating stroke unit was 9 [IQR 6–12]. The median time to v1 MRI was 30 days [IQR 18–37], the median lesion volume at v1 was 4.3 ml [IQR 1.2–23.4], and the median NIHSS at v1 was 4 [IQR 3–9]. At v2, the median time to MRI was 60 days [IQR 46–70], the median lesion volume was 3.0 ml [IQR 0.9–26.1], and the median NIHSS was 3 [IQR 2–5]. Patient characteristics of the entire cohort are described in Table 1.

**Table 1:** Patient demographics of the study cohort.

	**Total**
	**(*n* = 93)**
Age, mean (SD)	68.5 (11.3)
Female sex, % (*n*)	38 (35)
Cigarette smoking, % (*n*)	36 (33)
Hypertension, % (*n*)	82 (77)
DM, % (*n*)	29 (27)
Atrial fibrillation, % (*n*)	18 (17)
HLP,% (*n*)	52 (48)
i.v. thrombolysis, % (*n*)	32 (30)
Aerobic fitness training, % (*n*)	57 (53)
**TOAST criteria**	
Large artery atherosclerosis, % (*n*)	32 (30)
Cardioembolic, % (*n*)	28 (26)
Microangiopathic, % (*n*)	20 (19)
Others, % (*n*)	5 (5)
Undefined, % (*n*)	14 (13)
**Lesion volume (mL)**	
v1, median (IQR)	4.0 (1.2–23.4)
v2, median (IQR)	3.0 (0.9–26.1)
**NIHSS**	
Stroke Unit, median (IQR)	9 (6–12)
v1, median (IQR)	4 (3–9)
v2, median (IQR)	3 (2–5)
**Time to MRI in days**	
v1, median (IQR)	30 (18–37)
v2, median (IQR)	60 (46–70)

*DM, diabetes mellitus; HLP, hyperlipoproteinemia; i.v., intravenous; TOAST, Trial of ORG 10172 in Acute Stroke Treatment classification; NIHSS, National Institute of Health Stroke Scale*.

Eighty-eight patients (95%) underwent the v1 MRI with contrast agent application, compared to 70 patients at v2 (75%). Only 61% (*n* = 65) underwent an MRI with contrast agent application at both time points. Patient demographics of the analyzed cohort stratified by presence of increased BBBP at v1 and v2 are listed in Table 2.

**Table 2:** Patient demographics stratified by increased blood-brain barrier permeability (BBBP) at MRI before (v1) or after (v2) intervention.

	**Increased BBBP at v1**		***p*-value**	**Increased BBBP at v2**		***p*-value**
	**Yes (*n* = 70)**	**No (*n* = 18)**		**Yes (*n* = 52)**	**No (*n* = 18)**	
Age, mean (SD)	68.1 (11.4)	69 (11.0)	0.9^a^	67.4 (11.1)	70.4 (11.8)	0.6^a^
Female sex, % (*n*)	40 (28)	33 (6)	0.6^b^	39 (20)	22 (4)	0.2^b^
Cigarette smoking, % (*n*)	36 (25)	44 (8)	0.2^b^	35 (18)	22 (4)	0.2^b^
Hypertension, % (*n*)	87 (61)	72 (13)	0.1^b^	83 (43)	83 (15)	1.0^b^
DM, % (*n*)	27 (19)	28 (5)	0.3^b^	23 (12)	44 (8)	0.2^b^
Atrial fibrillation, % (*n*)	16 (11)	22 (4)	0.4^b^	14 (7)	22 (4)	0.4^b^
HLP,% (*n*)	47 (33)	72 (13)	0.1^b^	42 (22)	72 (13)	0.1^b^
i.v. thrombolysis, % (*n*)	36 (25)	17 (3)	0.6^b^	35 (18)	17 (3)	0.2^b^
Aerobic fitness training, % (*n*)	54 (38)	67 (12)	0.6^b^	58 (30)	56 (10)	1.0^b^
**TOAST criteria**			0.7^b^			0.4^b^
Large artery atherosclerosis, % (*n*)	33 (23)	28 (5)		31 (16)	33 (6)	
Cardioembolic, % (*n*)	27 (19)	28 (5)		27 (14)	28 (5)	
Microangiopathic, % (*n*)	20 (14)	28 (5)		25 (13)	17 (3)	
Others, % (*n*)	6 (4)	0 (0)		10 (5)	0 (0)	
Undefined, % (*n*)	14 (10)	17 (3)		8 (4)	22 (4)	
**Lesion volume (mL)**						
v1, median (IQR)	4 (1.7–26.1)	1 (0.4–7.1)	*0.01* ^c^	5 (1.4–26.1)	2 (0.4–7.2)	*0.03* ^c^
v2, median (IQR)	4 (0.9–27.8)	2 (0.3–10.0)	0.1^c^	5 (1.2–32.8)	1 (0.3–7.4)	*0.02* ^c^
**NIHSS**						
Stroke Unit, median (IQR)	9 (6–13)	9.5 (5–11)	0.5^c^	10 (6–12)	9 (5–11.25)	0.6^c^
v1, median (IQR)	5 (3–9)	3 (1.75–5.25)	*0.03* ^c^	5 (3–9)	3 (2–4.25)	*0.02* ^c^
v2, median (IQR)	3.5 (2–5)	2 (1–4.75)	*0.07* ^c^	4 (2–6)	2 (1–4)	*0.03* ^c^
**Time to MRI in days**						
v1, median (IQR)	26 (17–35)	34 (16.8–43.5)	0.1^c^	27 (19–35)	30 (12.5–44)	0.9^c^
v2, median (IQR)	57 (46–67)	64.5 (47.5–73.5)	0.3^c^	56 (46–67.25)	62.5 (39.5–72.25)	0.8^c^

*DM, diabetes mellitus; HLP, hypolipoproteinemia; i.v., intravenous; TOAST, Trial of ORG 10172 in Acute Stroke Treatment classification; NIHSS, National Institute of Health Stroke Scale*.

a *t-test*.

b *Chi^2^-test*.

c *Mann–Whitney U-Test*.

*Italic values emphasize the values that reached a significance levels/p-value ≤0.05*.

By visual assessment, increased BBBP on v1 MRI was observed in 80% of patients (*n* = 70), compared to 74% (*n* = 52) on v2 MRI. In patients with increased BBBP at v1, the median time to MRI was 26 days [IQR17–35]; in patients without an increased BBBP at v1, the median time to MRI was 34 days [IQR17–44]. In patients with increased BBBP at v2, the median time to MRI was 56 days [IQR 46–67.25]; in patients without an increased BBBP at v2, the median time to MRI was 63 days [IQR 40–72]. The majority of participants (84%, *n* = 78) demonstrated an increased BBBP on MRI at any time point, i.e., either at v1 or at v2 or at both time points. Only 16% (*n* = 15) of patients demonstrated no visible BBBP at all. Characteristics of patients stratified by presence of increased BBBP at any time vs. no increased BBBP at all are listed in Supplementary Table 2.

In final subtraction imaging across time points, the majority of cases (78%, *n* = 45) experienced a decrease in BBBP over time. In only three cases, an increase was detectable. Ten cases of BBBP increase remained unchanged over time. Examples of BBBP increase and decrease detection are presented in Supplementary Figures 1, 2.

### Patient Characteristics and BBBP

Visually assessed presence of increased BBBP at v1 was associated with larger median lesion volumes (4.4 ml [IQR 1.7–26.1] vs. 1.0 ml [IQR 0.4–7.1]; *p* = 0.01) and more severe stroke assessed by median NIHSS scores at v1 (5 [IQR 3–9] vs. 3 [IQR 2–5]; *p* = 0.03) in univariate analyses (Table 2). At v2, we could observe a similar association of visibly increased BBBP and larger median lesion volumes (4.7 ml [IQR 1.2–32.8] vs. 1.4 ml [IQR 0.3–7.4]; *p* = 0.02) and more severe strokes assessed by median NIHSS scores (4 [IQR 2–6] vs. 2 [IQR 1–4]; *p* = 0.03). There was no association between the presence of qualitatively increased BBBP and intravenous thrombolysis at v1 [36% (*n* = 25) vs. 17% (*n* = 3); *p* = 0.6] or v2 [35% (*n* = 18) vs. 17% (*n* = 3); *p* = 0.2, see Table 2]. Additionally, occurrence rates of increased BBBP were not significantly higher in the trial intervention group of aerobic fitness training at v2 (increased BBBP 58 vs. no increased BBBP 56%, see Table 2).

Further, increased BBBP was more frequently detected in females, both on v1 and v2. In additional exploratory *post-hoc* analysis, no independent association of female sex and increased BBBP at both time points could be observed (see Table 3). Here, prior history of hyperlipoproteinemia was observed to modify the risk of increased BBBP at v2 (OR 0.3, 95% CI 0.1–1.0; *p* = 0.04, see Table 3). After adjusting for pre-existing statin medication as secondary prevention post-stroke, pre-diagnosis of HLP was no longer significantly associated with lower risk of increased BBBP at v2 (OR 0.3 95% CI 0.1–1.0, *p* = 0.07).

**Table 3:** Multivariate regression analyses of factors associated with presence of increased BBBP before (v1) and after (v2) intervention.

	**Odds ratio (95% CI)**	***p*-value**
**Presence of increased BBBP v1**		
Female sex	1.3 (0.4–5.0)	0.7
Age	1.0 (0.9–1.0)	0.5
Arterial hypertension	6.0 (0.9–39.8)	0.1
Hyperlipoproteinemia	0.4 (0.1–1.5)	0.2
Lesion volume at v1	1.0 (1.0–1.1)	0.3
NIHSS at v1	1.1 (0.9–1.3)	0.5
**Presence of increased BBBP v2**		
Female sex	2.5 (0.7–9.8)	0.2
Age	1.0 (0.9–1.0)	0.4
Arterial hypertension	1.1 (0.2–7.2)	0.9
Hyperlipoproteinemia	0.3 (0.1–1.0)	*0.04*
Lesion volume at v2	1.0 (1.0–1.1)	0.4
NIHSS at v2	1.2 (0.9–1.6)	0.4

*Adjustment for variables which reached a significance level of p ≤ 0.05 in univariate analyses (lesion volume and severity of stroke based on NIHSS) and variables known from literature to influence the BBBP (age, sex, arterial hypertension and hyperlipoproteinemia). NIHSS, National Institute of Health Stroke Scale*.

*Italic values emphasize the values that reached a significance levels/p-value ≤0.05*.

In adjusted linear mixed models for the semi-quantitatively assessed intensity of increased BBBP (presented in Table 4), only the time from stroke onset to MRI was identified as a main effect and was inversely associated with increased BBBP [coefficient −0.002; Standard Error (SE) 0.007, *p* < 0.01]. Age, sex, and arterial hypertension were not identified as modifying factors.

**Table 4:** Linear mixed models for normalized contrast enhancement within region of interest (normalized CE-ROI) at MRI before (v1) and after (v2) intervention.

**Dependent variable: normalized CE-ROI**			
	**Fixed-effects**		
	**Coefficient**	**Std. Error**	***p*** **-value**
Time to MRI in days	−0.002	0.001	*0.001*
Time point of MRI (v1 vs. v2)	−0.009	0.026	0.724
Intervention group (training)	0.006	0.18	0.735
	**Random-effects**		
	**Estimate**	**Std. Error**	**95% CI**
Subject ID	0.044	0.018	0.020 − 0.096
Dependent variable: ^*^adjusted normalized CE-ROI			
	**Fixed-effects**		
	**Coefficient**	**Std. Error**	***p*** **-value**
Time to MRI in days	−0.002	0.001	*0.001*
Time point of MRI (v1 vs. v2)	−0.011	0.026	0.672
Intervention group (training)	0.005	0.018	0.776
Arterial hypertension	−0.028	0.026	0.297
Age	<0.001	0.001	0.945
Male sex	−0.006	0.018	0.761
	**Random-effects**		
	**Estimate**	**Std. Error**	**95% CI**
Subject ID	0.039	0.021	0.014–0.111

* *Adjusted for age, sex, and hypertension*.

*Italic values emphasize the values that reached a significance levels/p-value ≤0.05*.

Exploratory analysis showed that pre-existing atrial fibrillation was more frequently found in patients with persisting BBBP assessed by subtraction imaging (39 vs. 9%; *p* < 0.001). Concerning the trial intervention, we did not find differences between persisting and decreasing BBBP over time (54 vs. 58%, *p* = 0.8). Supplementary Table 3 summarizes the patient demographics stratified by evolution of BBBP at follow-up.

### Serum Biomarkers and BBBP

Due to the observation that hyperlipoproteinemia might influence the BBBP, we performed additional *post-hoc* analyses on low-density lipoprotein (LDL) and high-density lipoprotein (HDL): in univariate analysis, we observed a significant association between high LDL levels defined by laboratory cut-offs and an increased BBBP at v1 (78 vs. 56%; *p* = 0.01). Moreover, we found LDL at v1 as continuous (2.2 mmol/L [IQR 1.8–2.5] vs. 1.6 mmol/L [IQR 1.2–2.3]; *p* = 0.02) and dichotomized (76 vs. 40%; *p* < 0.01) variable to be associated with an increased BBBP at any time (see Supplementary Table 4). In multivariate analysis adjusted for age, sex, arterial hypertension, HLP, lesion volume, and stroke severity, high LDL levels were still significantly associated with the presence of increased BBBP at v2 (OR 4.4 95% CI 1.0–19.4, *p* = 0.03) and at any time (OR 4.9 95% CI 1.1–22.2, *p* = 0.02; see Table 5).

**Table 5:** Multivariate regression analyses of elevated low-density lipoprotein (LDL) levels before (v1) intervention with presence of increased BBBP before intervention and during follow-up.

	**Crude OR** **(95% CI)**	***p*-value**	Adjusted^*^ OR **(95%CI)**	***p*-value**
**Presence of increased BBBP v1**				
High LDL v1	2.8 (1.0–8.4)	0.06	2.1 (0.5–8.5)	0.3
**Presence of increased BBBP v2**				
High LDL v1	2.9 (0.9–9.1)	0.07	4.4 (1.0–19.4)	*<0.03*
**Presence of increased BBBP at any time**				
High LDL v1	4.8 (1.5–15.4)	*<0.01*	4.9 (1.1–22.2)	*<0.02*
**Increase/unchanged BBBP**				
High LDL v1	0.2 (0.02–1.7)	0.1	0.2 (0.02–1.9)	0.2

* *Adjusted for female sex, age, arterial hypertension, HLP, lesion volume, and NIHSS*.

*HLP, hyperlipoproteinemia; LDL, low-density lipoprotein; NIHSS, National Institute of Stroke Scale; OR, odds ratio; CI, confidence interval*.

*Italic values emphasize the values that reached a significance levels/p-value ≤0.05*.

High-sensitive CRP was available in 89 patients (96%), whereas TNFα and IL-6 values were available in 88 patients (95%) and 91 patients (98%), respectively. Levels of MMP-9 and VEGF were only available in a minority of blood samples (both *n* = 36, 39%). No differences between the levels of serum biomarkers (hs-CRP, TNFα, IL-6, VEGF, and MMP-9) as continuous and dichotomized variables before the intervention (v1) were observed in patients with or without increased BBBP neither at v1 nor at v2 (Table 6). Further, serum biomarker levels did not differ between patients with presence of increased BBBP at any time point and patients without increased BBBP at all (Supplementary Table 5). Moreover, there were no significant differences between the selected blood-derived biomarkers at v1 in patients with an increase of/unchanged BBBP and those with a decrease of BBBP over time (Supplementary Table 6).

**Table 6:** Serum biomarkers before (v1) intervention in patients with or without increased BBBP before and after (v2) intervention.

	**Total**	**Increased BBBP v1**		***p-*value**	**Increased BBBP v2**		***p-*value**
		**Yes**	**No**		**Yes**	**No**	
		**(*n* = 70)**	**(*n* = 18)**		**(*n* = 52)**	**(*n* = 18)**	
hsCRP mg/L, median (IQR)	4.8	2.6	1.6	0.4^a^	5.2	4.9	0.6^a^
	(1.2–10.6)	(1.1–7.8)	(0.5–8.5)		(1.7–10.8)	(0.9–17.6)	
TNFα pg/mL, median (IQR)	8.2	8.1	7.6	0.2^a^	7.7	7.6	0.5^a^
	(6.6–9.7)	(6.6–10.8)	(6.1–8.6)		(6.1–9.6)	(6.1–8.5)	
IL-6 pg/mL, median (IQR)	3.6	3.5	3.7	0.8^a^	3.6	3.4	1.0^a^
	(2.4–6.1)	(2.4–5.9)	(1.9–8.6)		(2.4–5.3)	(2.1–7.6)	
VEGF pg/mL, median (IQR)	718	718	855	0.3^a^	762	663	0.7^a^
	(433–1,070)	(436–1,025)	(549–1,397)		(433–1,070)	(341–1,203)	
MMP–9 ng/mL, median (IQR)	1,050	955	1,294	0.3^a^	1,092	942	0.6^a^
	(846–1,318)	(836–1,316)	(870–1,477)		(864–1,344)	(682–1,294)	
High hsCRP,% (*n*)	59.1	60	56	0.9^b^	64	56	0.7^b^
	(55)	(42)	(10)		(33)	(10)	
High TNFα, % (*n*)	48	49	33	*0.05* ^b^	46	39	0.3^b^
	(45)	(34)	(6)		(24)	(7)	
High IL–6, % (*n*)	43	41	44	0.8^b^	44	50	0.7^b^
	(40)	(29)	(8)		(23)	(9)	
High VEGF, % (*n*)	13	11	17	0.8^b^	15	11	0.6^b^
	(12)	(8)	(3)		(8)	(2)	
High MMP-9, % (*n*)	13	11	22	0.2^b^	14	11	1.0^b^
	(12)	(8)	(4)		(7)	(2)	

a *Mann–Whitney *U*-Test*.

b *Chi^2^-test*.

*Italic values emphasize the values that reached a significance levels/p-value ≤0.05*.

Differences in baseline characteristics between patients with and without elevated biomarkers levels are presented in Supplementary Table 7. As depicted in Supplementary Table 8, no association was observed between levels of serum biomarkers at v1 and BBB permeability at v1 or at v2 following adjustment for selected cerebrovascular risk factors.

### BBBP and Functional Outcome

At 6 months follow-up, 41% (*n* = 31) of patients had a favorable functional outcome (mRS 0–2) and 59% (*n* = 45) had an mRS ≥3. Neither the presence of increased BBBP on v1 (adjusted OR 0.9, 95% CI 0.6–1.4) nor the evolution of increased permeability (adjusted OR 1.1, 95% CI 1.0–1.3) was associated with favorable outcome 6 months after stroke (Table 7).

**Table 7:** Multivariate regression analyses of increased BBBP before intervention and during follow up and favorable functional outcome (mRS <3).

**Modified Rankin Scale: favorable outcome 6 months**		
	**Crude OR (95% CI)**	**Adjusted OR (95%CI)**
Presence of increased BBBP v1	1.1 (0.8–1.4)	0.9 (0.6–1.4)^a^
Presence of increased BBBP v2	1.0 (0.9–1.2)	0.9 (0.7–1.2)^b^
Increased BBBP at any time	0.7 (0.2–2.2)	0.2 (0.02–1.3)^c^
Increase/unchanged	1.1 (1.0–1.3)	1.1 (1.0–1.3)^d^
BBBP		

a *Adjusted for sex, age, and variables that reached a significance of ≤0.1 in univariate analysis (aHT arterial hypertension, HLP hyperlipoproteinemia, NIHSS National Institute of Health Stroke Scale at v1, lesion volume at v1, time to v1 MRI in days)*.

b *Adjusted for sex, age, and variables that reached a significance of ≤0.1 in univariate analysis (HLP hyperlipoproteinemia, NIHSS National Institute of Health Stroke Scale at v1, lesion volume at v1)*.

c *Adjusted for sex, age, and variables that reached a significance of ≤0.1 in univariate analysis (HLP hyperlipoproteinemia, NIHSS National Institute of Health Stroke Scale at v1, lesion volume at v1, time to v1 MRI in days)*.

d *Adjusted for sex, age, and variables that reached a significance of ≤0.1 in univariate analysis (atrial fibrillation)*.

*OR, odds ratio; CI, confidence interval*.

In *post-hoc* multivariate regression analyses using the median split (mRS 0–3: 68%, *n* = 52 vs. mRS 4–6: 32%, *n* = 24), we observed that a persisting BBBP in subtracted images was associated with worse functional outcome in patients at 6 months post-stroke (adjusted OR 1.2, 95% CI 1.0–1.4; *p* = 0.02).

## Discussion

In this exploratory analysis of the *BAPTISe* study, we observed that increased BBBP evaluated on contrast-enhanced MRI following moderate-to-severe ischemic stroke was detectable in approximately three out of four cases in early subacute phase of stroke. The presence and intensity of BBBP decreased over time; however, it remained detectable up to 2 months reflecting persisting BBB changes throughout recovery processes in the subacute phase post-stroke. Furthermore, we could demonstrate an association of an increased BBBP with larger lesion volumes and more severe strokes both before and after the trial intervention. To the best of our knowledge, this is the first clinical study to investigate the effect of increased BBBP on long-term outcome in subacute stroke patients.

Although increased BBBP assessed via contrast-enhanced MRI is frequently observed in the hyper-acute and acute phase following ischemia (3, 4, 33–36), the dynamic in later stages is far less understood. Several animal studies could demonstrate that an increased BBBP is still detectable up to 3 weeks following an ischemic event (3, 4). Previous clinical studies support the theory that BBBP may persist into subacute stages in some cases (33, 35, 36). In this study, we were able to observe that most stroke patients still demonstrated an increased BBBP of the ischemic lesion visible on contrast-enhanced MRI at 2 months after the acute event.

BBB dynamics are believed to be diverse and likely influenced by stroke severity (34). In our analyses, stroke severity defined by NIHSS was associated with the presence of an increased BBBP. This has not yet been observed in previous clinical BBBP studies (16–18); albeit BBBP was assessed using differing methodologies including CSF fluid-serum albumin ratios or CT-perfusion imaging in these studies. Moreover, in the current study, serially performed MRI allowed an evaluation of BBBP evolution over time. We observed that BBBP tended to decrease over time in this patient cohort. MR-based detection of increased BBBP might help to understand underlying regeneration mechanisms such as vascular remodeling and possible associations with functional outcome (24).

Whereas, previous studies have suggested that selected cytokines such as TNFα and IL-6 may serve as surrogate markers of increased BBBP (7, 8), we found no robust associations between selected pro-inflammatory biomarkers and BBB integrity in this patient cohort derived from a randomized-controlled stroke rehabilitation trial. Moreover, the levels of selected biomarkers representing pathophysiological mechanisms such as enzymatic proteolysis (MMP-9) and vascular remodeling (VEGF) did not differ between patients with and without increased BBBP.

One of our primary aims was to determine whether blood biomarkers could serve as surrogate markers of the presence and evolution of increased BBBP in subacute stroke. A handful of previously published studies described underlying associations between selected serum biomarkers and BBBP. For example, it has been shown that IL-6 increases BBB dysfunction in rodent models (7). Interestingly, it has been demonstrated that the inhibition of VEGF signaling diminishes the BBB impairment (37). This phenomenon was only observed in mice with pre-existing diabetes. However, in the current exploratory study, we found no robust associations between TNFα, hsCRP, IL-6, VEGF, and MMP-9 levels at v1 and the prevalence or evolution of BBBP in this cohort of subacute stroke patients.

We observed a higher rate of increased BBBP in patients with high LDL-cholesterol levels at inclusion (Table 5 and Supplementary Table 4). Our findings support the observation that elevated LDL levels influence the occurrence of an increased BBBP in the subacute stages of ischemic stroke. Cholesterol levels might influence BBB integrity through activation of inflammation and oxidative stress as described previously (38). Interestingly, previous clinical studies found that low LDL levels and low total cholesterol levels to be associated with higher rates of hemorrhagic transformation (39–41). Whether there is a causal connection between the effects of cholesterol levels on BBB integrity and increased risk of hemorrhagic transformation post-stroke should be investigated in detail in future, independent analyses.

Of note, preclinical studies have reported contradicting results. For example, Kalayci et al. (42) suggested that hypercholesterolemia might have a positive effect on the BBB by increasing the expression of tight junction proteins and thereby possibly decreasing paracellular permeability in rodents. Since the increased BBBP in the subacute stage of the ischemic event also represents regenerative processes, further studies on the lipoprotein's influence in the subacute setting may be of interest. Nevertheless, a comprehensive analysis in a larger independent cohort of stroke patients is warranted to support or refute the observations reported here.

Of note, due to the advantages and disadvantages of single biomarkers, there may be additional value of a combination of selected biomarkers for the prognostic value of increased BBBP. In their clinical study, Tu et al. (43) proclaimed that a panel of neuroendocrine biomarkers predicts functional outcome more efficiently than the NIHSS or single biomarkers alone, suggesting that certain biomarker panels may help in the early evaluation of stroke.

Previous studies suggest that the application of intravenous thrombolysis in the setting of acute stroke may contribute to BBB alteration due to its potential neurovascular toxicity and effect on neuro-inflammation following ischemia (44). However, we found no correlation between increased BBBP and intravenous thrombolysis in the current analysis.

The effect of training on the BBB is still under investigation; previous studies have implied that physical training might reduce oxidative stress and inflammatory processes and thereby strengthen the BBB integrity (20). In our study, a 4-week aerobic fitness training intervention was not identified as a modifier of BBBP over time (Table 2). These results are in line with the co-primary efficacy endpoints of the *PHYS-STROKE* trial. Here, no associations of the maximum walking speed and Barthel Index at 3 months post-stroke and the intervention were observed (29).

Assuming that BBBP affects the regenerative processes following ischemic tissue damage on a molecular basis, one might expect that long-lasting BBBP post-stroke may modify functional recovery (1). Previous studies that used imaging techniques as well as cerebrospinal fluid/serum albumin ratios to quantify the BBBP found that BBB alteration in the acute setting influenced patients' long-term outcome (14, 25, 26). Although in the current analysis, increased BBBP in subacute stages post-stroke were not associated with a favorable functional long-term outcome (mRS <3), we observed an association between a persisting BBBP and worse functional outcome (mRS >3). However, these findings should be validated in larger, independent cohort analyses. Detection of persisting BBBP in subacute stages could, for example, guide individual rehabilitation strategies (45, 46) if the studies find prognostic value in prolonged BBBP. The identification of a surrogate marker of BBBP (i.e., via increase in selected serum biomarkers) would provide an accessible tool to easily assess BBBP in the clinical setting and subsequently guide further therapeutic procedures.

In summary, this is the first study to show increased BBBP on contrast-enhanced MRI through early and late subacute stages of stroke, which takes us closer to understanding the complex regeneration and recovery processes taking place after ischemia (24, 32). A deeper knowledge of the prognostic value of patient-individual BBBP dynamics in subacute stages may guide therapeutic and rehabilitation strategies in the future.

## Limitations

This analysis has several limitations that warrant discussion. First, this is an exploratory analysis as part of a randomized-controlled stroke rehabilitation trial. Therefore, the existing MRI protocol was not explicitly designed for BBB evaluation. However, dynamic contrast enhanced-MRI is the most well-established method for the visualization and quantification of BBBP (30, 47, 48) and contrast-enhanced MRI is a reliable method to assess BBB integrity and has been successfully applied in previous clinical studies (5, 31). Of note, the time point of MR-imaging at v1 and v2 was inhomogeneous, causing overlap, and limiting comparability across individuals with differing imaging time points. Furthermore, we used subtraction maps to show increased BBBP changes within one subject over time (49, 50); the limitations of this technique include complex post-processing steps and the final visual evaluation of the subtraction images, which is subject to rater-bias. Lastly, the number of participants who received a contrast agent application at both v1 and v2 MRI time points was low, minimizing the sample size considerably and increasing the risk of Type II errors. Concerning the blood-based biomarkers, the total numbers of MMP9 and VEGF measurements were likewise considerably low, which also limits the statistical power of this analysis. Of note, there are several well-known biomarkers of inflammation influencing the BBBP in subacute stroke such as IL-1beta and IL-8 (51, 52). Although these cytokines were not included in the current analysis, they could add diagnostic value in further analyses.

## Conclusion

The permeability of the blood-brain barrier assessed on contrast-enhanced MRI decreases during the subacute phase of ischemic stroke but remains detectable for up to 2 months in three out of four patients after moderate-to-severe ischemic stroke. The presence of increased BBBP is associated with more severe strokes and larger infarct volumes. We did not observe a relation between the presence of increased BBBP and exposure to aerobic fitness training. In our cohort, we could not detect an association between the selected serum biomarkers (hs-CRP, TNFα, IL-6, MMP-9, and VEGF) and the phenomenon of an increased BBBP. No clear associations were observed between an increased BBBP and functional outcome at 6 months post-stroke; hence, the long-term prognostic value of the phenomenon in subacute phases of an ischemic stroke remains unclear and warrants further investigation in independent cohorts.

## Data Availability Statement

The raw data supporting the conclusions of this article will be made available by the authors, without undue reservation.

## Ethics Statement

The studies involving human participants were reviewed and approved by Institutional review board of Charité-Universitätsmedizin Berlin (EA1/137/13 and EA1/138/13). The patients/participants provided their written informed consent to participate in this study.

## Author Contributions

AF, MEb, AN, and TR conceived or designed the study and supervised the study. SM was responsible for analyzing the data and drafted the manuscript. AK and SP contributed in statistical analysis. AF, MEb, and MEn obtained funding. AN attests that all listed authors meet authorship criteria and that no others meeting the criteria have been omitted. All authors critically revised the manuscript for important intellectual content and gave final approval of the version to be published.

## Funding

This trial was supported by the German Ministry for Health and Education (01EO0801) through Center for Stroke Research Berlin grant G.2.15. The funder had no role in study design; data collection, analysis, or interpretation; or writing the manuscript. AN was participant in the BIH-Charité Clinician Scientist Program funded by the Charité –Universitätsmedizin Berlin and the Berlin Institute of Health. MEn received funding from DFG under Germany's Excellence Strategy—EXC-2049–390688087, BMBF, DZNE, DZHK, EU, Corona Foundation, and Fondation Leducq.

## Conflict of Interest

MEn reports grants from Bayer and fees paid to the Charité from AstraZeneca, Bayer, Boehringer Ingelheim, BMS, Daiichi Sankyo, Amgen, GSK, Sanofi, Covidien, Novartis, and Pfizer, all outside the submitted work. JF reports consulting and advisory board fees from Abbvie, AC Immune, Artemida, BioClinica, Biogen, BMS, Brainomix, Cerevast, Daiichi-Sankyo, EISAI, F. Hoffmann-La Roche AG, Eli Lilly, Guerbet, Ionis Pharmaceuticals, IQVIA, Janssen, Julius Clinical, jung diagnostics, Lysogene, Merck, Nicolab, Premier Research, and Tau Rx, outside the submitted work. The remaining authors declare that the research was conducted in the absence of any commercial or financial relationships that could be construed as a potential conflict of interest.

## Publisher's Note

All claims expressed in this article are solely those of the authors and do not necessarily represent those of their affiliated organizations, or those of the publisher, the editors and the reviewers. Any product that may be evaluated in this article, or claim that may be made by its manufacturer, is not guaranteed or endorsed by the publisher.
