# Supplementary material for: Evolution of Blood-Brain Barrier Permeability in Subacute Ischemic Stroke and Associations With Serum Biomarkers and Functional Outcome
Source: Front Neurol. 2021 Oct 20;12:730923. doi: 10.3389/fneur.2021.730923 (PMC8567961; doi:10.3389/fneur.2021.730923)
Supplement: Supplementary file 1 [file Data_Sheet_1.pdf]

SUPPLEMENTAL FILES

Evolution of blood brain barrier permeability in subacute ischemic stroke and associations with serum biomarkers and functional outcome

Sarah Müller, Anna Kufner, Torsten Rackoll, Andrea Dell Orco, Ralf Mekle, Sophie K. Piper, Jochen B. Fiebach, Kersten Villringer, Agnes Flöel, Matthias Endres, Martin Ebinger, and Alexander H. Nave

Figures and Tables

Table S1: Inclusion and Exclusion Criteria for the BAPTISe trial (NCT01954797)

Table S2: Patients demographics stratified by increased blood brain barrier permeability (BBBP) at any time point and no increased BBB permeability at all

Table S3: Patients demographics stratified by evolution of BBBP (increase/unchanged vs decrease)

Table S4: Serum biomarkers before intervention (v1) stratified by presence of increased BBBP before intervention and during follow up

Table S5: Serum Biomarkers before (v1) intervention in patients with increased BBBP at any time point or patients without increased BBBP

Table S6: Serum Biomarkers before (v1) intervention in patients with increase of/unchanged BBBP or decrease of BBBP

Table S7: Patients demographics stratified by subgroups with High hsCRP, TNFα, IL-6, VEGF and MMP-9 at v1 (Yes vs No)

Table S8: Blood-biomarkers levels before (v1) intervention and presence of increased BBBP before and after (v2) intervention

Figure S1: Magnet resonance T1-imaging - Increase of blood brain barrier permeability (BBBP)

Figure S2: Magnet resonance T1-imaging - Decrease of blood brain barrier permeability (BBBP)

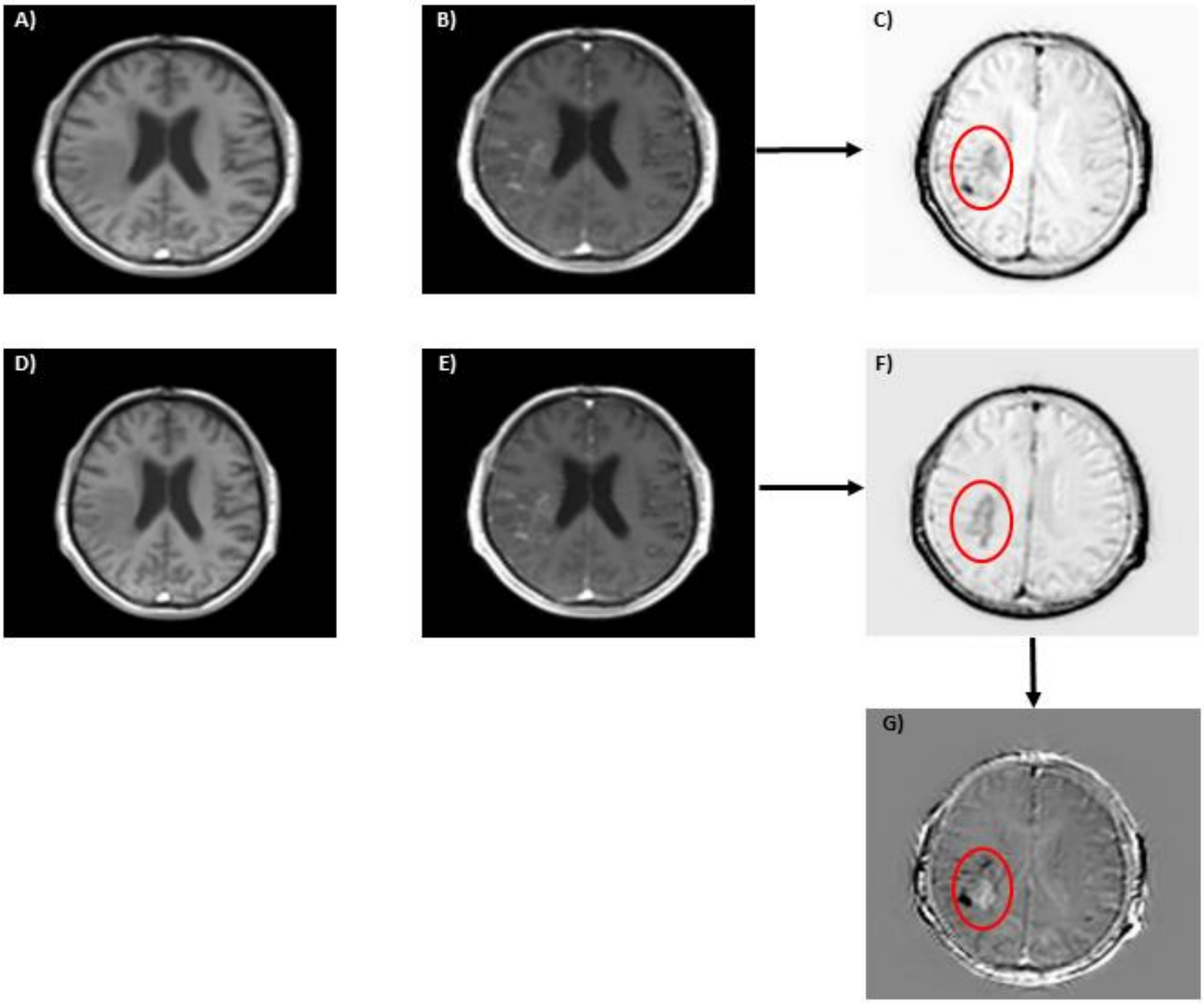

Figure S1. Magnet resonance T1-imaging - Increase of blood brain barrier permeability (BBBP) over time (Middle cerebral artery infarction right hemisphere)**:**
Before intervention v1: A) v1 pre-contrast agent (CA), **B)** v1 post-CA, **C)** v1 subtraction pre-post CA: hypo intensity (red circle);
After intervention v2: D) v2 pre-CA, **E)** v2 post-CA, **F)** v2 subtraction pre-post CA: hypo intensity (red circle);
G) final subtraction v1-v2: hyper intensity (red circle)

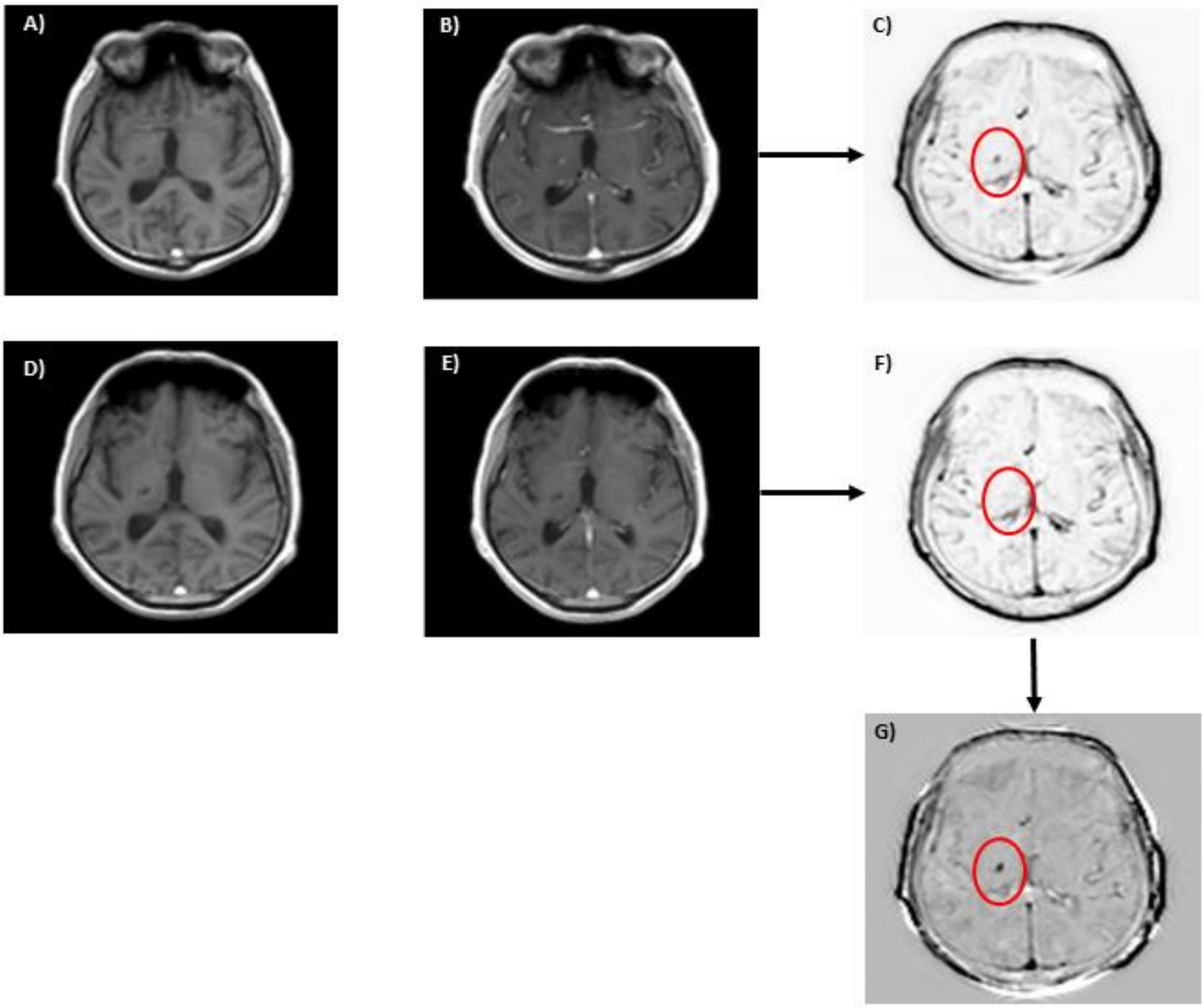

Figure S2. Magnet resonance T1-imaging - Decrease of blood brain barrier permeability (BBBP) over time (Anterior choroid artery right hemisphere):

Before intervention v1: A) v1 pre-contrast agent (CA), **B)** v1 post-CA, **C)** v1 subtraction pre-post CA: hypo intensity (red circle);

After intervention v2: D) v2 pre-CA, **2E)** v2 post-CA, **F)** v2 subtraction pre-post CA: iso intensity (red circle);

G) final subtraction v1-v2: hypo intensity (red circle)

Table S1: Inclusion and Exclusion Criteria for the *BAPTISe* trial (NCT01954797)

**Inclusion Criteria**
Age: > 18 years
Diagnosis of subacute ischemic stroke (within 5-45 days after stroke onset)as determined by initial MRI/CT scan of the brain
Cortical, sub-cortical, or brainstem affection
Barthel Index (BI) <65 at inclusion
Able to sit for at least 30 seconds (unsupported or supported, i.e., holding onto supports such as the edge of the bed)
Ability to perform aerobic exercise, determined by by responsible physician
Provision of written consent
**Exclusion criteria**
Lacking ability to comply with study requirements
Stroke due to intracranial haemorrhage
Previous subarachnoid hemorrhage or other hemorrhagic stroke
Progressive stroke
Not able to receive magnetic resonance imaging scans, including perfusion imaging
Unable to perform the required exercises due to medical, musculoskeletal, or neurological problems
Required help of at least 1 person to walk before stroke due to neurological (e. g., advanced Parkinson's disease, Amyotrophic Lateral Sclerosis, Multiple Sclerosis) or non-neurological co-morbidities (e. g. heart failure, orthopaedic problems)
Life expectancy < 1 year as determined by responsible physician
Drug or alcohol addiction within the last six months
Significant current psychiatric illness defined as medication-refractory of bipolar affective disorder, psychosis, schizophrenia or suicidality
Current participation in another interventional trial

Table S2: Patients demographics stratified by increased blood brain barrier permeability (BBBP) at any time point and no increased BBB permeability at all

	BBBP increased at any time point	BBBP not increased at all	*p-value*
	**84% (n=78)**	**16% (n=15)**	
Age, mean (SD)	68.1 (11.5)	70.4 (10.1)	0.9 [a]
Female sex, % (n)	40 (31)	27 (4)	0.3 [b]
Cigarette smoking, % (n)	36 (28)	33 (5)	0.9 [b]
Hypertension, % (n)	83 (65)	80 (12)	0.4 [b]
DM, % (n)	27 (21)	40 (6)	0.3 [b]
Atrial fibrillation, % (n)	17 (13)	27 (4)	0.4 [b]
HLP,% (n)	46 (36)	80 (12)	*0.02* [b]
Cardiovascular disease, % (n)	10 (8)	7 (1)	0.7 [b]
Previous stroke, % (n)	15 (12)	20 (3)	0.7 [b]
i.v. thrombolysis, % (n)	36 (28)	13 (2)	0.2 [b]
Aerobic fitness training, % (n)	56 (44)	60 (9)	0.8 [b]
SUE, % (n)	23 (18)	33 (5)	0.4 [b]
**TOAST criteria**			0.8 [b]
large artery atherosclerosis, % (n)	33 (26)	27 (4)	
cardioembolic, % (n)	27 (21)	33 (5)	
microangiopathic, % (n)	21 (16)	20 (3)	
others, % (n)	6 (5)	0 (0)	
undefined, % (n)	13 (10)	20 (3)	
**Lesion volume (mL)**			
v1, median (IQR)	4.6 (1.7-26.1)	0.7 (0.3-6.7)	*0.01* [c]
v2, median (IQR)	4.2 (1.0-28.6)	1.3 (0.2-8.0)	*0.07* [c]
**NIHSS**			
Stroke Unit, median (IQR)	10 (6-13)	8 (5-11)	0.2 [c]
v1, median (IQR)	5 (3-9)	3 (2-5)	*0.04* [c]
v2, median (IQR)	4 (2-5.75)	2 (1-4.5)	*0.09* [c]
**Time to MRI in days**			
v1, median (IQR)	28.5 (17.75-35.5)	39 (29.25-44)	*0.02* [c]
v2, median (IQR)	58 (46-70)	70 (55.5-74.5)	*0.03* [c]

DM: diabetes mellitus, HLP: hyperlipoproteinemia, i.v.: intravenous, TOAST: Trial of ORG 10172 in Acute Stroke Treatment classification, SUE: severe unexpected events, NIHSS: National Institute of Health Stroke Scale. Cardiovascular disease is defined by prior history of myocardial infarction, coronary heart disease or peripheral artery occlusive disease.

[a:] t-test; [b:] Chi²test, [c:] MannWhitney-U Test.

Table S3: Patients demographics stratified by evolution of BBBP (increase/unchanged vs decrease)

	**BBBP Increase/unchanged (22%, n=13)**	**BBBP Decrease (78%, n=45)**	***p-value***
Age, mean (SD)	69.5 (10.5)	66.4 (11.6)	0.8 [a]
Female sex, % (n)	31 (4)	36 (16)	0.8 [b]
Cigarette smoking, % (n)	46 (6)	36 (16)	0.5 [b]
Hypertension, % (n)	77 (10)	84 (38)	0.5 [b]
DM, % (n)	15 (2)	28 (13)	0.3 [b]
Atrial fibrillation, % (n)	39 (5)	9 (4)	*<0.001* [b]
HLP,% (n)	62 (8)	47 (21)	0.4 [b]
Cardiovascular disease, % (n)	39 (5)	20 (9)	0.3 [b]
Previous stroke, % (n)	23 (3)	16 (7)	0.5 [b]
i.v. thrombolysis, % (n)	31 (4)	27 (12)	0.8 [b]
Aerobic fitness training, % (n)	54 (7)	58 (26)	0.8 [b]
SUE, % (n)	8 (1)	24 (11)	0.2 [b]
**TOAST criteria**			0.5 [b]
large artery atherosclerosis, % (n)	23 (3)	36 (16)	
cardioembolic, % (n)	39 (5)	20 (9)	
microangiopathic, % (n)	23 (3)	24 (11)	
others, % (n)	0 (0)	9 (4)	
undefined, % (n)	15 (2)	11 (5)	
Lesion volume (mL)			
v1, median (IQR)	4.2 (0.8-7.1)	4.6 (1.1-25.7)	0.4 [c]
v2, median (IQR)	4.3 (0.8-17.9)	3.1 (0.7-26.1)	0.8 [c]
**NIHSS**			
Stroke Unit, median (IQR)	10 (5.5-11)	9 (5-12)	0.7 [c]
v1, median (IQR)	3 (2-7.5)	4 (3-7.75)	0.3 [c]
v2, median (IQR)	3 (0.5-5.5)	3 (2-5)	0.5 [c]
**Time to MRI in days**			
v1, median (IQR)	25 (12.5-44.5)	24 (16.25-34.74)	0.8 [c]
v2, median (IQR)	54 (42-72)	53 (41-66)	0.3 [c]

DM: diabetes mellitus, HLP: hyperlipoproteinemia, i.v. intravenous, TOAST: Trial of ORG 10172 in Acute Stroke Treatment classification, SUE: severe unexpected events, NIHSS: National Institute of Health Stroke Scale. Cardiovascular disease is defined by prior history of myocardial infarction, coronary heart disease or peripheral artery occlusive disease.

[a:] t-test; [b:] Chi^{2}test;[c:] MannWhitney-U Test.

Table S4: Serum biomarkers before intervention (v1) stratified by presence of increased BBBP before intervention and during follow up

	**Total**	**Increased BBBP v1**		***p-value***	**Increased BBBP v2**		***p-value***	**Increased BBBP at any time**	**No increased BBBP at all**	***p-value***	**BBBP Increase/unchanged**	**BBBP Decrease**	***p-value***
	**(n=91)**	**Yes (n=68)**	**No (n=18)**		**Yes (n=51)**	**No (n=18)**		**(n=76)**	**(n=15)**		**(n=13)**	**(n=44)**	
LDL mmol/L, median (IQR)	2.1 (1.6-2.5)	2.1 (1.8-2.5)	1.9 (1.4-2.5)	0.2^{c}	2.2 (1.8-2.6)	1.8 (1.5-2.3)	0.07^{c}	2.2 (1.8-2.5)	1.6 (1.2-2.3)	*0.02^{c}*	2.4 (1.8-2.6)	2.1 (1.6-2.5)	0.3^{c}
High LDL, % (n)	70 (64)	78 (53)	56 (10)	*0.01^{b}*	78 (40)	56 (10)	0.1^{b}	76 (58)	40 (6)	*$<0.01^{b}$*	92 (12)	71 (31)	0.1^{b}
HDL mmol/L, median (IQR)	1.2 (1.0-1.5)	1.2 (1.0-1.4)	1.3 (1.1-1.5)	0.4^{c}	1.2 (1.0-1.5)	1.1 (1.0-1.4)	0.8^{c}	1.2 (1.0-1.5)	1.3 (1.0-1.5)	0.7^{c}	1.4 (1.1-1.4)	1.2 (1.0-1.4)	0.3^{c}
High HDL, % (n)	42 (38)	38 (26)	50 (9)	0.5^{b}	39 (20)	39 (7)	0.7^{b}	40 (30)	53 (8)	0.3^{b}	54 (7)	34 (15)	0.3^{b}

High Low density Lipoprotein (LDL) is defined as ≥1.8 mmol/L, High High density Lipoprotein (HDL) is defined as ≥1.3 mmol/L. b: Chi^{2}test;c: MannWhitney-U Test.

Table S5: Serum Biomarkers before (v1) intervention in patients with increased BBBP at any time point or patients without increased BBBP

	**Increased BBBP at any time (84%, n=78)**	**No increased BBBP at all (16%, n=15)**	***p-value***
hsCRP mg/L, median (IQR)	5.0 (1.7-10.5)	3.9 (0.8-14.7)	0.4 [c]
TNFα pg/mL, median (IQR)	8.2 (6.7-10.4)	7.7 (6.2.9.1)	0.3 [c]
IL-6 pg/mL, median (IQR)	3.6 (2.4-5.2)	3.8 (2.2-12.9)	0.7 [c]
VEGF pg/mL, median (IQR)	734 (439-1070)	663 (341-1343)	1.0 [c]
MMP-9 ng/mL, median (IQR)	1045 (848.5-1317)	1077 (682-1330)	1.0 [c]
High hsCRP,% (n)	60 (47)	53 (8)	0.5 [b]
High TNFα, % (n)	50 (39)	40 (6)	0.7 [b]
High IL-6, % (n)	41 (32)	53 (8)	0.4 [b]
High VEGF, % (n)	13 (10)	13 (2)	0.8 [b]
High MMP-9, % (n)	11.5 (9)	20 (3)	0.6 [b]

High high sensitive C-reactive protein (hsCRP) is defined as ≥3,0 mg/L, high tumornecrosis factor alpha (TNFα) as ≥8,1 pg/mL, high interleukin 6 (IL-6)as ≥3,6 pg/mL, high vascular endothelial growth factor (VEGF) as ≥991 pg/mL and high matrix metallopeptidase 9 (MMP9) as ≥1279 ng/mL.; [b:] Chi^2test;[c:] MannWhitney-U Test.

Table S6: Serum Biomarkers before (v1) intervention in patients with increase of/unchanged BBBP or decrease of BBBP

	**BBBP Increase/unchanged (22%, n=13)**	**BBBP Decrease (78%, n=45)**	***p-value***
hsCRP mg/L, median (IQR)	7.2 (0.9-14.2)	4.9 (1.7-13.1)	0.9 [c]
TNFα pg/mL, median (IQR)	8.1 (6.4-9.9)	7.3 (6.0-9.1)	0.4 [c]
IL-6 pg/mL, median (IQR)	3.6 (2.1-7.2)	3.5 (2.3-5.9)	0.8 [c]
VEGF pg/mL, median (IQR)	1010 (499-1293)	717,5 (466.8-956.8)	0.4 [c]
MMP-9 ng/mL, median (IQR)	1077 (887-1434)	1070 (632.5-1344.3)	0.8 [c]
High hsCRP,% (n)	62 (8)	60 (27)	0.6 [b]
High TNFα, % (n)	54 (7)	32 (15)	0.2 [b]
High IL-6, % (n)	39 (5)	44 (20)	0.5 [b]
High VEGF, % (n)	11 (8)	17 (3)	0.8 [b]
High MMP-9, % (n)	11 (8)	22 (4)	0.2 [b]

High high sensitive C-reactive protein (hsCRP) is defined as ≥3,0 mg/L, high tumornecrosis factor alpha (TNFα) as ≥8,1 pg/mL, high interleukin 6 (IL-6)as ≥3,6 pg/mL, high vascular endothelial growth factor (VEGF) as ≥991 pg/mL and high matrix metallopeptidase 9 (MMP9) as ≥1279 ng/mL. [b:] Chi^2test;[c:] MannWhitney-U Test.

Table S7: Patients demographics stratified by subgroups with High hsCRP, TNFα, IL-6, VEGF and MMP-9 at v1 (Yes vs No)

	High hsCRP		*p-value*	High TNFα		*p-value*	High IL-6		*p-value*	High VEGF		*p-value*	High MMP-9		*p-value*
	**Yes (n=55)**	**No (n=34)**		**Yes, (n=45)**	**No, (n=43)**		**Yes (n=12)**	**No (n=24)**		**Yes, (n=12)**	**No, (n=24)**		**Yes (n=40)**	**No (n=51)**	
Age, mean (SD)	68.3 (11.4)	68.6 (10.8)	0.6[a]	71.6 (11.5)	66.1 (10.5)	0.2[a]	68.8 (11.6)	68.6 (11.9)	0.5[a]	66.9 (12.5)	69.5 (11.4)	0.3[a]	73.2 (9.3)	65.3 (11.5)	0.4
Female sex, % (n)	40 (22)	32 (11)	0.5[b]	36 (16)	40 (17)	0.7[b]	17 (2)	38 (9)	0.2[b]	8 (1)	42 (10)	*0.04*	35 (14)	39 (20)	0.7
Cigarette smoking, % (n)	38 (21)	32 (11)	0.6[b]	29 (13)	42 (18)	0.2[b]	17 (2)	33 (8)	0.3[b]	50 (6)	17 (4)	*0.04*	38 (15)	33 (17)	0.7
Hypertension, % (n)	84 (46)	85 (29)	0.8[b]	87 (39)	81 (35)	0.5[b]	83 (10)	88 (21)	0.7[b]	83 (10)	88 (21)	0.7	83 (33)	86 (44)	0.6
DM, % (n)	18 (10)	47 (16)	*<0.01*[b]	29 (13)	28 (12)	0.9[b]	8 (1)	46 (11)	*0.03*[b]	17 (2)	42 (10)	0.1	25 (10)	33 (17)	0.4
Atrial fibrillation, % (n)	15 (8)	24 (8)	0.3[b]	29 (13)	9 (4)	*0.02*[b]	8 (1)	21 (5)	0.3[b]	8 (1)	21 (5)	0.3	23 (9)	16 (8)	0.4
HLP, % (n)	49 (27)	56 (19)	0.5[b]	36 (16)	63 (27)	*0.01*[b]	42 (5)	46 (11)	0.8[b]	42 (5)	46 (11)	0.8	48 (19)	53 (27)	0.6

High high sensitive C-reactive protein (hsCRP) is defined as ≥3,0 mg/L, high tumornecrosis factor alpha (TNFα) as ≥8,1 pg/mL, high interleukin 6 (IL-6)as ≥3,6 pg/mL, high vascular endothelial growth factor (VEGF) as ≥991 pg/mL and high matrix metallopeptidase 9 (MMP-9) as ≥1279 ng/mL. DM: Diabetes mellitus, HLP: hyperlipoproteinemia. [a:] t-test; [b:] Chi^2test;[c:] MannWhitney-U Test.

Table S8: Blood-biomarkers levels before (v1) intervention and presence of increased BBBP before and after (v2) intervention

	Crude OR (95% CI)	Adjusted* OR (95%CI)
**Presence of increased BBBP v1**		
High hsCRP v1	1.4 (0.5-4.0)	1.4 (0.5-4.3)
High TNFα v1	1.7 (0.6-5.3)	2.1 (0.6-7.2)
High IL-6 v1	0.9 (0.3-2.7)	1.0 (0.3-3.0)
High VEGF v1	0.6 (0.1-3.3)	0.4 (0.1-3.3)
High MMP v1	0.3 (0.1-1.9)	0.2 (0.01-2.6)
**Presence of increased BBBP v2**		
High hsCRP v1	1.6 (0.5-4.7)	1.0 (0.3-3.6)
High TNFα v1	1.1 (0.4-3.5)	1.9 (0.5-6.7)
High IL-6 v1	0.8 (0.3-2.4)	1.2 (0.3-3.9)
High VEGF v1	1.7 (0.3-10.8)	0.8 (0.1-8.2)
High MMP v1	1.4 (0.2-8.8)	0.3 (0.02-5.3)

*adjusted for age, sex, and variables that reached a significance of ≤0.1 in univariate analyses.
High high sensitive C-reactive protein (hsCRP) is defined as ≥3,0 mg/L, high tumornecrosis factor alpha (TNFα) as ≥8,1 pg/mL, high interleukin 6 (IL-6)as ≥3,6 pg/mL, high vascular endothelial growth factor (VEGF) as ≥991 pg/mL and high matrix metallopeptidase 9 (MMP-9) as ≥1279 ng/mL. OR: odds ratio.
